# Low prostaglandin-endoperoxide synthase-2 gene expression in colorectal carcinomas may predict poorer survival

**DOI:** 10.3332/ecancer.2024.1814

**Published:** 2024-12-06

**Authors:** Uchenna Simon Ezenkwa, Sebastian Anebuokhae Omenai, Oluwadamilare Iyapo, Chinedu Anthony Ezekekwu, Adesoji E Adetona, Chima Uzoma Akunwata, Ayotunde Oladunmi Ale, Henry Okwuchukwu Ebili

**Affiliations:** 1 Department of Histopathology, Federal University of Health Sciences Azare, Azare 751101, Bauchi, Nigeria; 2 Department of Pathology, Federal Medical Centre Azare, Azare 751101, Bauchi, Nigeria; 3 Department of Anatomical Pathology, Edo State University Uzairue, Auchi 312002, Edo, Nigeria; 4 Department of Pathology, Federal Medical Centre, Ebute Metta, Lagos 101212, Nigeria; 5 Bristol Haematology and Oncology Centre, University Hospital, Bristol and Weston NHS Trust, Bristol BS28ED, UK; 6 Department of Morbid Anatomy & Histopathology, Olabisi Onabanjo University, Ago-Iwoye 121101, Ogun State, Nigeria; 7 Department of Haematology, University College Hospital Ibadan, Ibadan 200221, Oyo, Nigeria; 8 Department of Medicine, Olabisi Onabanjo University/University Teaching Hospital, Sagamu 121101, Ogun, Nigeria; a https://orcid.org/0000-0002-7022-8268; b https://orcid.org/0000-0002-1841-1487; c http://orcid.org/0000-0003-1102-094X; d https://orcid.org/0000-0003-3703-2316; e https://orcid.org/0000-0002-7548-5087; f https://orcid.org/0000-ooo3-1779-7311; g https://orcid.org/0000-0002-2135-1796

**Keywords:** prostaglandin-endoperoxide synthase-2, cyclooxygenase-2, colorectal carcinoma, TCGA, prognosis, survival

## Abstract

**Introduction:**

Prostaglandin-endoperoxide synthase-2 (ptgs2), otherwise called Cyclooxygenase 2, is overexpressed in colorectal carcinoma (CRC) compared to normal tissues. However, the impact of differential expression among ptgs2-positive tumours on CRC prognosis has not been well investigated. By sub-stratifying positive tumour expression, this study determined its potential influence on patients’ outcomes.

**Methods:**

The Cancer Genome Atlas database was explored to determine CRC cases with RNA-Sequence (RNA-Seq) transcript data and matched clinicopathological data alongside gene copy number variation and methylation status. Descriptive, chi-square, Fisher exact, Linear-by-Linear associations, logistic and Kaplan-Meier statistics were used to determine proportions, associations, predictors and survival between ptgs2 and tumour parameters using Statistical Package for Social Sciences version 20. Two-tailed *p*-value <0.05 was accepted as statistically significant.

**Results:**

There were 534 CRC classified predominantly as adenocarcinoma not otherwise specified (86.3%) and mucinous carcinoma (12.4) histologically included in this study. Marker (ptgs2) expression ranged from 0.02 FPKM-131.89 FPKM, (Median 1.4 FPKM). The majority of the cases (53.4%) were diagnosed at an early stage and showed high ptgs2 RNA-Sequence (RNA-seq) expression in 51.5% (275/534). Significant associations were seen between ptgs2 expression and histological subtype (*p* < 0.001), lymphovascular invasion (p = 0.013), pN2 stage (> 6 positive lymph nodes) (*p* = 0.011) and American Joint Committee on Cancer Staging stage (*p* = 0.028), and these all had lower ptgs2 expression. On regression analysis, histological differentiation emerged as a predictor of ptgs2 expression (Odds ratio 2.749, 95% confidence interval 1.479–5.108, *p* < 0.001). Also, gene methylation was associated with reduced ptgs2 expression. Overall survival was significantly inferior among individuals with low ptgs2 tumours (*p* = 0.018) while that for disease-free survival was non-significant (*p* = 0.327).

**Conclusion:**

CRCs with low ptgs2 transcripts are associated with poorer survival. This finding suggests a need for closer follow up and tailored adjuvant therapy for these patients.

## Introduction

Colorectal carcinoma (CRC) is the most common malignancy of the gastrointestinal tract [1]. Global estimates suggest that there were about 1.9 million new CRC cases and 904,000 deaths in 2022, ranking third in terms of incidence but second in terms of mortality from all cancers [2]. This high burden is disproportionately distributed between developed and developing countries, the former having higher incidence while the latter has higher burden of mortality rates, respectively [1, 3]. Alcohol consumption, tobacco smoking, consumption of red or processed meat and increase in body fatness raise the risk of developing the disease while calcium supplements, whole grains, fiber and dairy products consumption, as well as physical activity, are considered protective, particularly for colon cancer [4]. The increasing adoption of cancer-promoting lifestyles and less protective ones, especially among low- and middle-income countries shows that the burden of the disease is bound to increase [2]. To mitigate this impact, there is a need to refine our understanding of the disease process for a more effective preventive or treatment approach.

Carcinogenesis is a multistage process characterised by cumulative genetic alterations in cellular homeostatic replicating pathways [5]. In the colorectum, for example, it has been shown that gain-of-function changes in genes promoting or loss-of-function in those suppressing cell proliferation are fundamental to tumourigenesis [6]. Usually, the transformation of normal epithelium to carcinoma begins with the dysregulation of the canonical Wnt pathway mediated by a mutated APC gene and gives rise to a dysplastic mucosa (adenoma) [7]. Subsequent accumulation of other genetic defects such as SMAD4/2, hTERT and TP53 lead to advanced adenoma and carcinoma. Other established pathways include loss of DNA mismatch repair capability, chromosomal instability (resulting from aneuploidy, deletions, insertions, amplifications or loss of heterozygosity), microsatellite instability, serrated adenoma pathway (activating V600E mutation in BRAF gene) and DNA methylation [8].

Recent studies suggest that synergistic pathways involving genes regulating the tumour microenvironment, histone acetylation and inflammation can also influence the carcinoma process. Perturbations in COL11A1 and HDAC9 are believed to induce disturbances in some signaling pathways such as the RTK-RAS-PI3K, Wnt, TGF-β and TP53 in the colon, thereby influencing cancer development [9, 10]. Toll-like receptor signaling in the colon has also been implicated in tumourigenesis through the induction of chronic inflammation within the gut [11]. Indeed, signature genes responsible for the progression to CRC in patients with ulcerative colitis have been proposed, including WFDC2, TTLL12, THRA, EPHB3 [12], ARID1A, FBXW7, KRAS, RNF, APC, P53 and SMAD4 genes [13]. Thus, these indicate a putative role of inflammatory mediators in carcinogenesis [12].

One such inflammatory mediator is prostaglandin E2, a byproduct of cyclooxygenase-2 (COX-2) enzyme action on arachidonic acids [14]. In 1994, Eberhart *et al* [15] described a differential overexpression of COX-2 mRNA in CRC and adenomatous polyps compared to normal tissues and proposed that COX-2 could be a control target for CRC [15]. Subsequent studies investigating this observation have reached varying conclusions, a situation largely due to differing methods of detecting and/or scoring the marker expression [16]. For example, Sheehan *et al* [17] found heterogenous tumour staining with COX 2 antibodies and further showed that more low-grade tumours with COX-2 positivity had better overall survival than high-grade tumours [17]. Likewise, Ogino *et al* [18] using immunohistochemistry, showed that COX-2 positive patients had inferior colon cancer-specific mortality even after adjusting for the stage and clinical characteristics of the patients [18]. On the other hand, Zahedi *et al* [19] also observed that COX-2 expression by tumour cells had no influence on tumour invasiveness or patient outcome, even though tumour tissues had higher COX-2 expression compared to normal tissues. As such, they opined that iCOX-2 plays a role in initiating the carcinogenic process but not its progression [19].

The varying mutational landscapes illustrated above show that CRC is a heterogenous disease, and although much is known about it, its 5-year survival is still below optimum despite therapies informed by these advances. There is therefore a need to interrogate further what is known in order to gain more insights that can help improve outcomes. Most studies investigating the role of ptgs2 (COX-2) in CRC have focused on differential expression between normal and malignant tissues or between marker-expressing and non-expressing tumours. This presupposes that tumours expressing ptgs2 behave alike irrespective of the degree to which each tumour expresses the marker. However, while evidence supports the hypothesis that COX-2 expression among COX-2-expressing tumours is not uniform [17], the potential influence of this variation on the prognosis and clinicopathological features of CRC has not been sufficiently investigated. The present study relied on ptgs2 transcripts data from The Cancer Genome Atlas (TCGA) database, to determine the association between high and low ptgs2 expression and patient survival as a primary endpoint, and by extension its relationship with clinicopathological factors.

## Materials and methods

RNA-Seq data of CRCs stored as Colorectal Adenocarcinoma (TCGA, PanCancer Atlas) was downloaded from TCGA repository at cBioportal [20]. Additionally, data on copy number variation (CNV) and methylation status of the genes were also retrieved. The procedure for extracting such data is well explained by Gao *et al* [21]. Matched patient clinical and tumour histopathological information such as gender, age at diagnosis, tumour location within the large bowel (colon or rectum), tumour histological subtype, tumour depth (pT), tumour lymph node involvement, number of positive lymph nodes (pN), tumour metastasis, lymphovascular invasion, vascular permeation and tumour staging according to the American Joint Committee on Cancer Staging, overall and disease-free survival (DFS) in months were extracted too. Having a documented ptgs2 RNA gene transcripts were mandatory for inclusion into the study while cases without documented ptgs2 transcripts were excluded. The data used in this study was derived from a published source with complete participant de-identification, and therefore does not require ethical review or approval.

### Data analysis

Statistical analysis was applied to the obtained data using the Statistical Package for Social Sciences version 20. Association between ptgs2 expression level and overall survival was the primary endpoint while DFS and association with overall American Joint Committee on Cancer Staging (AJCC) tumour stage were secondary endpoints. Categorical variables were summarized as proportions while continuous variables were described as median using descriptive statistical tool. The association between ptgs2 overexpression and the clinicopathological parameters was tested using Pearson’s chi-square, Fisher exact test or Linear-by-Linear associations. Also, the association between ptgs2 expression and ptgs2 CNV was evaluated using linear-by-linear association statistics, while the relationship between ptgs2 and methylation status was determined through Spearman correlation analysis. Binomial logistic regression was used to test for predictors of ptgs2 expression while Kaplan-Meier statistics was applied to the data to test for association between ptgs2 and overall and DFS. All *p*-value were determined at a two-tailed significance level of <0.05. Outputs were presented as text, tables and figures.

## Results

There were 629 entries for CRC on the dataset out of which, 95 (15.1%) had no ptgs2 data and were excluded from the study. Five hundred and thirty-four (84.9%) cases comprising of 285 males and 249 females with a median age of 68 years (age range 31–90 years) were therefore included in the study. Table 1 shows the patient and tumour pathological characteristics. The tumours were broadly categorised as adenocarcinoma not otherwise specified (86.3%) and mucinous carcinoma (12.4%). Most of the tumours were located within the colon (83%) and the majority were seen at an early stage (AJCC I-IIA).

### PTGS2 gene expression pattern

The dichotomised ptgs2 expression profile showed RNA overexpression in 51.5% (275) of the CRCs. The median expression was 1.4 FPKM ranging from 0.02 to 131.89 FPKM. Table 2 shows associations between ptgs2 expression and the tumour characteristics. Mucinous carcinomas were more likely to express high ptgs2 compared to adenocarcinomas (*p* < 0.001). Other observed significant associations were between ptgs2 expression and lymphovascular invasion (*p* = 0.013), lymph node metastasis (*p* = 0.011) and tumour stage (*p* = 0.028). The majority of tumours with lymphovascular invasion by tumour cells had low ptgs2 expression (105/191; 55%). In contrast, tumours without lymphovascular invasion had higher ptgs2 (163/288, 56.6%). Likewise, CRC cases having early AJCC tumour staging (I-IIA) showed higher ptgs2 compared to late-stage tumours (AJCC IIB – IV). Tests of association between ptgs2 and vascular, pM and pT staging did not reach statistical significance (Table 2).

### Logistic regression for predictive markers of ptgs2 expression

The result of the statistical test for predictors of ptgs2 expression in CRC using binomial logistic regression is as shown in Table 3. Only the histological subtype significantly predicted COX-2 gene expression (*p* < 0.001). AJCC tumour stage, lymphovascular invasion and lymph node metastasis status did not reach acceptable level of statistical significance (p > 0.05).

### CNV, methylation status and ptgs2 expression

There was available CNV data for 524 tumour tissues. COX-2 gene deletions and amplifications were seen in 50 (9.54%) and 117 (22.33%) cases, respectively, while wild-type variants were 357 (68.13%). The association between CNV and ptgs2 expression was statistically non-significant.

The correlation between ptgs2 and overall methylation status was both inverse and significant (*r* = −0.289; *p* < 0.001). Methylation analysis involved several loci, with some of these loci identified in as few as 156 tumour tissue samples, while others were detected in up to 516 cases. Only loci expression data present for up to a minimum of 300 patients was included in this analysis. Most of the loci had an inverse relationship with ptgs2 expression. These include COX2_cg00690431 (*r* = −0.098; *p* = 0.076), COX2_cg04881125 (*r* = −0.081; *p* = 0.142), COX2_cg07422329 (*r* = −0.168; *p* = 0.002), COX2_cg08482694 (*r* = −0.102; *p* = 0.021), COX2_cg10180406 (*r* = −0.201; *p* < 0.001), COX2_cg13986130 (*r* = −0.067; *p* = 0.126), COX2_cg16101346 (*r* = −0.311; *p* < 0.001), COX2_cg18335243 (*r* = −0.090; *p* = 0.041), COX2_cg22365834 (*r* = −0.058; *p* = 0.310), COX2_cg23070111 (*r* = −0.126; *p* = 0.022), COX2_cg24887140 (*r* = 0.173; *p* = 0.002), COX2_cg25147026 (*r* = −0.331; *p* < 0.001), COX2_cg25837803 (*r* = −0.235; *p* < 0.001) and COX2_cg26564040 (*r* = −0.245; *p* < 0.001). Two loci showed positive correlations and included COX2_cg07422329 (*r* = 0.388; *p* < 0.001) and COX2_cg17419623 (*r* = 0.070; *p* = 0.205).

**Figure 1.**
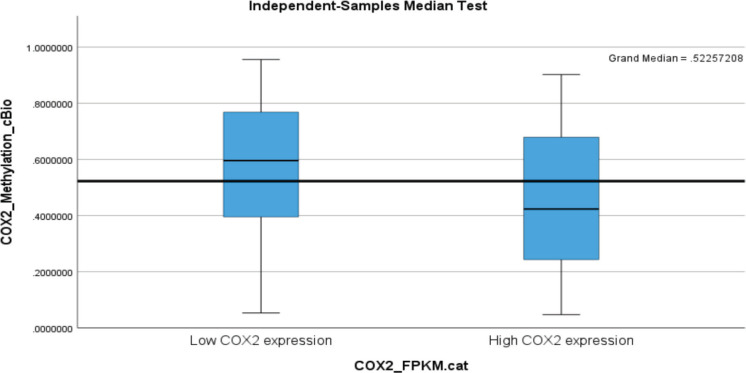
A box plot showing higher PTGS2 (COX-2) methylation status in low PTGS2-expressing colorectal carcinoma with its median value being above the *overall median of 0.52*.

Segregating between low and high ptgs2 expression, there was a statistically significant difference in the median methylation status (expression) between low and high ptgs2 expressing tumours. Low ptgs2 CRC had higher gene methylation compared to high ptgs2 CRC (Figure 1). Sustained significant differential methylation between low and high ptgs2-expressing tumours was observed at loci COX2_cg07422329 (*p* < 0.001), COX2_cg08482694 (*p* = 0.036), COX2_cg16101346 (*p* < 0.001), COX2_cg18335243 (*p* = 0.028), COX2_cg24887140 (*p* = 0.003) and COX2_cg25147026 (*p* < 0.001).

### Ptgs2 expression and survival outcomes

**Figure 2.**
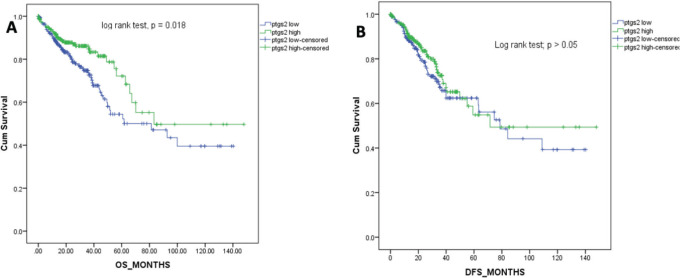
Kaplan-Meier survival curves for the overall survival (a) and DFS (b) in months.

The overall survival for the patients ranged from 0 to 148 months with a median of 21.1 months. Figure 2 shows the Kaplan-Meier survival curve for the overall survival of the patients included in this study. Patients with higher ptgs2 expression had better survival outcomes than those with low ptgs2. The Log Rank (Mantel-Cox) test for survival outcome was statistically significant (Figure 2A). The median DFS for this population was 20 (0–148) months. Low ptgs2 tumours had poorer DFS although there was no significant difference in the DFS between the two groups as shown by the Log Rank (Mantel-Cox) test (*p* = 0.327, Figure 2B).

## Discussion

The findings of this study showed that CRC tumours expressing ptgs2 do not all behave similarly. In addition, those with lower marker expression had significantly poorer overall survival. Also, although DFS was not significantly different between ptgs2-low and high tumours, ptgs2-low tumour patients had inferior DFS compared to ptgs2-high tumour patients. Few authors have documented similar findings in colorectal and breast cancers previously [22, 23]. Kim *et al* [22] showed that CRC patients with elevated COX-2 expression experienced favourable recurrence-free survival compared to COX-2-low tumours with overexpressing cancers having the late onset of recurrence [22]. Similarly, recent meta-analysis data have demonstrated better overall and disease-specific survival in high COX-2-expressing CRC [24, 25]. In contrast, Wu and Sun [26], Kosumi *et al* [27] and Mima *et al* [28] found higher mortality among high COX-2 expressing tumour patients in their respective studies. Notably, these studies classified both low and negative COX-2-expressing tumours in one category, [26–28] whereas the present study focused only on tumours with ptgs2 transcripts. More studies are however needed to further test these observations and determine other meaningful ways of interpreting the biomarker in CRC.

Notwithstanding these observed differences, our result has implications for CRC research and care. Evidence shows that the use of anti-COX-2 agents improves disease-specific survival in CRC patients post-diagnosis [25]. We believe that the potential benefit of COX-2 inhibition would apply to both low- and high-ptgs2 tumours, although, patients with low ptgs2 tumours may require a closer follow-up and individualized adjuvant therapy to control their disease. This highlights a need to sub-categorise ptgs2 expression in CRC tumours such that low marker tumours are delineated from high marker tumours and also from those in which the marker is absent or undetected, and testing their prognostic relationships in future study designs.

Whilst lower CRC disease survival among low-ptgs2-tumours was unexpected, it nevertheless offers insights into the various ways by which the emerging role of the biomarker can be interrogated. First, the results showed that early-stage tumours, those without lymphovascular invasion and those having fewer numbers of lymph node metastasis, had higher ptgs2 expression than advanced tumours. This suggests that the biomarker may have been produced at a higher magnitude during tumor initiation, potentially playing a role in promoting the early stages of tumor development [16, 19]. There are literatures supporting this position. Ptgs2 is an inducible gene and was shown at its primary characterization to be induced by the mitogen Rous Sarcoma Virus with its product, PGE2, mediating tumour cell proliferation [29, 30]. Its (ptgs2) expression has been shown to promote, while its inhibition prevents colorectal adenomas [30, 31]. Williams *et al* [32] showed from their experiment through COX-2 inhibition, that COX-2 mediates transitions in the cell cycle by lifting G2/M blockade. Other researchers have also observed that COX-2 promotes cell proliferation in CRC by up-regulating micro-RNA miR-21, an antagonist of PGE2-degrading enzymes and tumour suppressor genes 15-PGDH and PDCD4 [33]. Likewise, COX-2 cross-talk with p53 led to inverse reciprocal regulation that reduced p53-mediated apoptosis in CRC in another study [34]. These observations therefore suggest a role for ptgs2 in initiating colorectal carcinogenesis [16].

Second, the differential expression of higher ptgs2 among early-stage tumours could support an alternative role as a tumour suppressor. From our result, the diminished ptgs2 expression seen with more invasive tumour characteristics may suggest that the marker expression is suppressed from its initial rate of production as the tumour advances. This loss-of-function could therefore lead to a more aggressive disease phenotype leading to worse overall survival among these patients. The ptgs2 methylation data also showed that the gene expression reduced in proportion to the methylation status, suggesting ptgs2 methylation as a regulatory mechanism in CRC. One mechanism by which ptgs2 could influence tumour suppression is through interaction with other mediators. Studies have shown that ptgs2 overexpression up regulates Kruppel-like factor-4 (KLF4), a tumour suppressor gene that mediates apoptosis in dividing cells but is lost in CRC [35, 36]. Thus, loss or downregulation of ptgs2 could potentially lead to dysregulation of KLF4 and similar mediators, leading to poor disease outcomes.

Another plausible explanation relates to synergistic interaction among ptgs2, tumour pathological factors and other molecular mediators. For example, tumour stage is an established independent prognostic factor in CRC [37] and likely played the most significant role in this study. Also, the interplay between ptgs2 and other well-known molecular markers of CRC prognosis has become a subject of recent investigation among researchers. Increased COX-2 is associated with increased matrix metalloproteinase-7 synthesis in colonic polyps, thus preparing the ground for invasiveness; post-diagnosis aspirin intake showed more benefit in tumours with coexisting high COX-2 and mutated PIK3C gene; COX-2-derived PGE2 induces PD-1 expression by CRC cells thereby evading host immune system; while inhibition of COX-2 re-sensitises resistant Epidermal Growth Factor Receptor (EGFR) receptors in CRC and increases tumour cell death by down-regulating miR-21 with elevated PDCD4 (a tumour suppressor) and 15-PGDH (a PGE2 degrading enzyme); to mention a few [38–43]. Therefore, the overall interpretation of the present study should be done without overlooking the roles these mediators and tumour histopathological characteristics could play in the disease progression and outcome.

Further on the histopathological variables, we found significantly elevated ptgs2 in mucinous carcinomas and this was sustained on logistic regression. Mucinous CRC are known to have high PIK3C, microsatellite instability (MSI) and BRAF mutations and are more susceptible to PD-1 inhibition [44]. They also tend to influence poorer survival although those with high MSI status show better outcomes [45, 46]. How this interacts to influence tumour progression needs to be elucidated. However, we think that the higher ptgs2 observed among mucinous carcinomas could be as a result of the stimulating effect of mucin on ptgs2 expression in the tumour microenvironment that results in the upregulation of these genes, further suggesting a role for inflammatory mediators such as COX-2 in tumour progression [47]. Therefore, higher ptgs2 levels in mucinous carcinoma, lower amounts in more invasive cancers, together with the influence of tumour staging gives us confidence that indeed, ptgs2 expression in this study had prognostic value.

There were several limitations to this study. First, the limited number of studies investigating the prognostic impact of the PTGS2 (COX-2) gene expression in marker-expressing colorectal cancers (CRCs) made it challenging to compare our findings with existing literature. This scarcity of data highlights the need for more comprehensive research in this area. Second, it was not feasible to determine whether patients had prior exposure to COX-2 inhibitors before surgery, a factor that could potentially influence PTGS2 expression and affect our observations. This limitation could be addressed in future studies by incorporating patient histories regarding COX-2 inhibitor use into a prospective study design. Overall, further research is necessary to explore the hypothesis that positive ptgs2 expression or its product, COX-2, in CRC should be further stratified to enhance prognostic benefit in patient management, as we suggest in our findings.

## Conclusion

Sub-categorising CRC tumours expressing ptgs2 into low and high expressors showed a significant effect on overall survival in this study. This suggests that all marker-expressing tumours do not behave alike and thus, may need to be treated differently. While this could suggest novel approaches in CRC therapy, caution is rather advised in interpreting these findings without more studies.

## Conflicts of interest

The authors have no relevant financial or non-financial interests to disclose.

## Funding

The authors declare that no funding or other form of support was received for this study.

## Consent to participate

Not required.

## Consent to publish

Not required.

## Ethics approval

The data used in this study are available on the public domain, hence no ethical approval was required.

## Author contributions

All authors contributed to the study conception and design. Material preparation, data collection and analysis were performed by USE, HOE, SAO and AOA. The first draft of the manuscript was written by USE and all authors commented on previous versions of the manuscript. All authors read and approved the final manuscript.

## Data availability

The datasets generated and/or analysed during the current study are available on the cBioportal repository (cBioportal.org).

## Figures and Tables

**Table 1.** Clinicopathological features of the study population.

Variable (*N*)	Frequency (*f*)	Percentage (%)
Sex Male Female	285 249	63.4 46.6
Tumour location Colon Rectum Missing	445 86 3	83.3 16.1 0.6
Histological diagnosis Adenocarcinoma Mucinous carcinoma Missing	461 66 7	86.3 12.4 1.3
Vascular invasion No Yes Missing	344 115 75	64.4 21.5 14.0
Lymphovascular invasion No Yes Missing	288 191 55	53.9 35.8 10.3
AJCC stage I IIA IIB and stage IIC III IV Missing	95 193 16 153 73 4	17.8 36.1 3.0 28.7 13.7 0.7

**Table 2.** Association between ptgs2 expression pattern and colorectal cancer pathological characteristics.

Variable	Ptgs2 expression		*p* value
	**Low**	**High**	
Tumour location Colon Rectum	213 46	232 40	0.340
Histological diagnosis Adenocarcinoma Mucinous carcinoma	237 20	224 46	0.001*
Lymphovascular invasion Yes No	105 125	86 163	0.013*
Lymph node metastasis N0 N1 N2	142 60 57	171 68 35	0.011*
Distant metastasis M0 M1	187 43	210 31	0.082
pTstage T1–2 T3–4	50 209	56 218	0.743
Vascular invasion Yes No	57 162	58 182	0.646
AJCC stage I IIA IIB and IIC III IV	40 91 5 79 43	55 102 11 74 30	0.028*

* Significant value at *p* < 0.05

**Table 3.** Binomial logistic regression assessing predictors of ptgs2 expression in CRC.

Variable	Category	Exp(B)	95% Confidence interval	*p* value
AJCC stage	Reference (stage I) IIA IIB and IIC III IV	0.776 1.575 0.172 0.129	0.451–1.335 0.489–5.070 0.017–1.777 0.014–1.212	0.359 0.446 0.140 0.073
Lymphovascular invasion	Ref (No) Yes	0.697	0.454–1.069	0.098
Histological diagnosis	Ref (Adenocarcinoma) Mucinous carcinoma	2.749	1.479–5.108	0.001*
Lymph node involvement	Ref (N0) N1 N2	5.517 3.691	0.559–54.425 0.374–36.411	0.144 0.264

* Significant *p* value *p* < 0.05; CI confidence interval
